# Surgical management for an infected urachal cyst in an adult: Case report and literature review^☆^

**DOI:** 10.1016/j.ijscr.2019.03.041

**Published:** 2019-03-30

**Authors:** Adel Elkbuli, Kyle Kinslow, John D. Ehrhardt, Shaikh Hai, Mark McKenney, Dessy Boneva

**Affiliations:** a Department of Surgery, Kendall Regional Medical Center, Miami, FL, United States; b University of South Florida, Tampa, FL, United States

**Keywords:** CT, computed tomography, Urachal remnants, Urachal cyst infection, Two-stage approach, Cysto-urachal malignancy

## Abstract

**Introduction:**

Incomplete urachal obliteration during fetal development gives rise to distinct malformations of the median umbilical ligament. Most anomalies are asymptomatic and resolve during early infancy, but some go unrecognized until adulthood. These rare cases can present with acute abdominal symptomatology secondary to infected urachal remnants.

**Presentation of case:**

A 20-year-old man presented with periumbilical pain. Physical exam showed a warm, erythematous infra-umbilical mass that was tender to palpation. CT revealed an infected urachal cyst. The patient underwent urachal abscess incision and drainage with cyst excision. The patient returned home on postoperative day two. Two-week outpatient follow-up confirmed an uncomplicated recovery.

**Discussion:**

Surgical excision of urachal anomalies in adults is curative and preventive against recurrent infection and malignancy, but preoperative management is currently unstandardized. Current literature recommends a two-stage approach characterized by preoperative antibiotics and cyst incision and drainage followed later by complete surgical excision. Our patient underwent a single therapeutic approach with preoperative antibiotics and definitive operative excision. Our patient had a comparable outcome with no complications and a short hospitalization.

**Conclusion:**

Preoperative antibiotics with excision of urachal remnants was effective in our case. Staged approaches are appropriate in complicated cases as a means to reduce post-operative infection rates and hospital stays. Urachal remnant excision is recommended in adults to prevent future malignancy.

## Highlights

• Case report of an umbilical urachal cyst presenting as an infected umbilical hernia.
• Congenital abnormalities such as these may present as a periumbilical soft tissue infection.
• Complete excision of the urachal cyst for pathologic examination is recommended.
• Patients can be successfully treated with short course of antibiotics and total excision.

## 1 Introduction

During early embryologic development, the urinary bladder is continuous with the allantois, a canalized fibromuscular stalk that connects the fetal bladder to the umbilical cord for drainage. The urachal canal normally obliterates as the bladder descends into the fetal pelvis, forming a fibrous connection with the ventral abdominal wall known as the median umbilical ligament. Complete obliteration typically occurs during late fetal development or early infancy (<6 months) [1].

When urachal obliteration fails, four distinct embryologic malformations can result: patent urachus, umbilical-urachal sinus, vesicourachal diverticulum, or urachal cyst. In patent urachus, the urachal canal remains open and drains the urinary bladder into the umbilicus. Those with umbilical-urachal sinus possess a single blind opening that may drain into the umbilicus. Patients with a vesicourachal diverticulum have urachal tissue that remains patent inferiorly and creates an outpouching from the dome of the urinary bladder. The process of urachal obliteration can result in a retained urachal cyst along the median umbilical ligament [2].

Many of the aforementioned entities are diagnosed during early infancy and monitored with ultrasonography [3,4]. After two years, as many as 80% of defects resolve, with some remaining who become surgical candidates because of urachal remnant infections. When symptomatic, children can present with fever, umbilical drainage, and a tender infra-umbilical mass [5]. Albeit rare, urachal remnants can go unrecognized until adulthood and come to clinical attention with acute abdominal symptomatology resembling appendicitis, Meckel’s diverticulum, or incarcerated hernia [6]. In this setting, CT imaging is sensitive for identifying urachal anomalies and assessing their spatial relationship with the urinary bladder and neighboring structures. Early recognition and treatment for infected urachal remnants lowers the risk for fistula formation or rupture, both of which can elicit peritonitis, abscess, and sepsis.

Herein, we report a case of an infected urachal cyst in a 20-year-old man discovered on CT scan and treated with preoperative antibiotics and operative therapy. We also discuss the literature for surgical management of urachal disease and associated complications. This case has been reported in line with the SCARE criteria [7].

## 2 Case presentation

A 20-year-old man presented to the emergency department after four days of progressively worsening periumbilical pain. He was moving heavy boxes for his job when he began experiencing pain and was unable to finish his work. He reported pain with defecation but denied fever, chills, nausea, emesis, weight loss, and recent travel or illness. Past surgical history included branchial cleft cyst excision as a child. On abdominal exam, a one square-centimeter erythematous infra-umbilical mass was exquisitely tender to palpation. Laboratory data on admission demonstrated a WBC count of 10.7 × 10^3^ cells/μL and urinalysis was unremarkable. Based on history and physical exam, the patient underwent diagnostic evaluation for suspected incarcerated umbilical hernia.

**Fig. 1:**
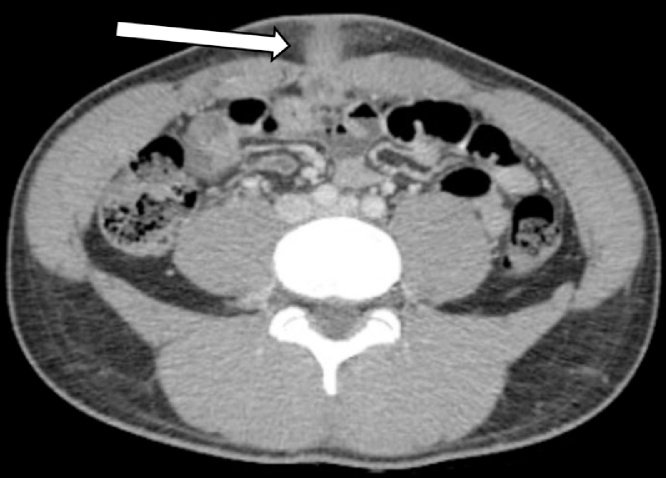
Axial CT abdomen showing urachal remnant extending through the ventral abdominal wall with associated inflammatory infiltrate and central lucency suggestive of abscess.

**Fig. 2:**
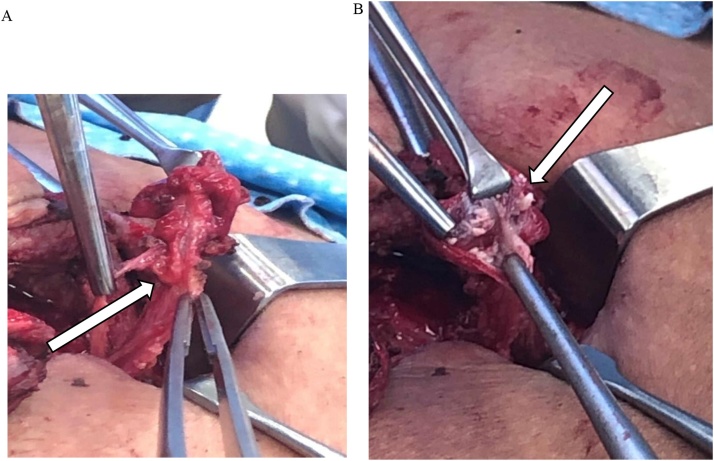
**A**. Intraoperative urachal cyst excision via median mini-laparotomy. **B**. Intraoperative expression of sebaceous material from infected urachal cyst.

**Fig. 3:**
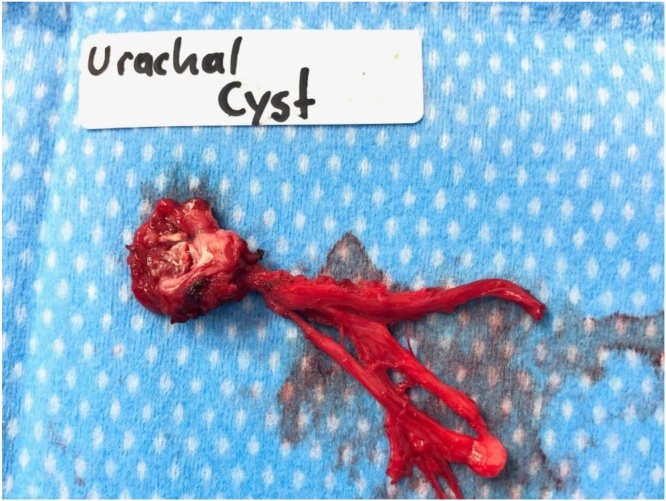
Excised urachal cyst specimen.

CT abdomen/pelvis revealed a four-centimeter segment of organized periumbilical inflammation with central lucency passing the ventral abdominal wall into the anterior abdominal compartment (Fig. 1). The process was extraperitoneal and there was no evidence of communication with the urinary bladder. These findings were consistent with an inflamed urachal remnant complicated by abscess. Our patient received intravenous antibiotics in preparation for an operation. The following day he underwent abscess incision and drainage followed immediately by urachal cyst excision through a four-centimeter infra-umbilical midline mini-laparotomy. The urachal cyst and remnants were dissected inferiorly to confirm no communication with the urinary bladder before total excision (Fig. 2A). Investigation of the cyst contents revealed white sebaceous material (Fig. 2B). Pathology examined the 4 × 3 x 0.7-centimeter segment of fibromembranous tissue and confirmed intraoperative impressions of the specimen (Fig. 3).

The patient was admitted to the surgical floor where he noted his pain was markedly improved. The next day he was discharged to home on post-operative day two with adequate pain control. Two-week follow up in the outpatient surgery clinic confirmed an uncomplicated recovery.

## 3 Discussion

Urachal cyst infections predispose patients to a number of acute complications, including bladder fistula formation, cyst rupture, peritonitis, and sepsis. Surgical excision of embryologic remnants is the therapeutic mainstay for symptomatic urachal disease, but preoperative management strategies often vary. Some advocate for a two-stage management approach focused on treating the urachal cyst infection with antibiotics and ultrasound-guided drainage before operative excision [[8], [9], [10]]. Others report removal of the infected urachal remnants without allowing time for infection and inflammation to subside [8,11]. The two-stage approach emphasizes infection resolution before surgical intervention as superior for reducing the risk of postoperative complications (wound infection and urine leak) and shortening the average length of hospitalization (5.8 vs. 9.2 days) when compared to surgery alone, but the sample sizes described were small [9,11]. One case managed a urachal cyst infection with two weeks of broad-spectrum antibiotic therapy before operative excision, altogether resolving the infection and forgoing drainage of cyst contents [5].

Our patient received antibiotics and prompt surgical excision without experiencing any postoperative infectious complications. His two-day hospitalization was shorter than the average reported with the two-stage management described above. Our approach was nearly identical to a case published in emergency medicine literature that also yielded a good outcome [12]. Factors that contribute to expedient recovery include early detection of urachal cyst infections, extent and severity of cyst infection, and sensitivity of the inciting pathogen to antibiotic therapy. A two-stage approach may be more appropriate when managing cases complicated by fistula, cyst rupture, purulent urachal sinus drainage, widespread abscess, and sepsis.

Some urachal cyst infections in pediatric patients have been successfully managed with more conservative measures. In a fifteen-patient case series of symptomatic urachal anomalies, three infected urachal cysts were treated with antibiotics and percutaneous drainage (two cases) or laparoscopic drainage (one case) without total excision. The patients in this case series were an average of 3.5 years-old, ranging from four weeks to fourteen-years-old. Urachal infection resolved in all cases, and complete urachal obliteration was confirmed with ultrasonography at a mean follow up of 26 months. These findings suggest that draining infected urachal cysts may be sufficient for the remnants to completely obliterate thereafter. Although symptomatic children can undergo cyst drainage and expect spontaneous urachal obliteration, this remains unclear for adults [13].

Another variable influencing surgical management for urachal disease is the perceived risk of future malignancy. Urachal carcinoma is a rare neoplasm with poor prognosis believed to arise from residual epithelia within urachal malformations from fetal development. Evidence from pediatric surgical literature suggests that asymptomatic anomalies in children do not confer significant risk for malignancy because the vast majority spontaneously resolve with time [14]. The same cannot be said for adults, where an investigation of 130 adult cases from 1951 to 2005 revealed that 51% of urachal specimens were malignant [15]. The most common features associated with these cases were patients over the age of 55 and those who presented with hematuria. Considering that most urachal disease presents in pediatric patients, our 20-year-old patient is on the older end of the spectrum for urachal disease. Percutaneous or laparoscopic drainage may have resolved the acute infection, but the relationship between sonographic disappearance of cysts and complete obliteration of primitive urachal remnants has not been established. For these reasons, we believe the evidence for non-operative management alone is lacking for preventing future neoplastic complications in adults.

Our patient’s presentation and physical exam suggested umbilical hernia as the predominant pathology before imaging revealed an infected urachal cyst. The patient was not septic and operative drainage and excision was successful. Follow up in the office showed no untoward outcomes.

## 4 Conclusion

We report a case of urachal cyst infection with associated umbilical hernia in a 20-year-old man. He received IV antibiotics and underwent successful urachal cyst excision, local abscess I&D, and primary umbilical hernia repair the following day. After urinary retention resolved on postoperative day one, he was discharged on postoperative day two and made an uncomplicated recovery. Current literature provides evidence to support a two-stage treatment approach as most effective in reducing post-operative complications and prolonged hospital stay. However, our case demonstrated that short term administration of IV antibiotics prior to complete surgical excision was sufficient with no postoperative complications or prolonged hospital stay. This approach can be considered safe with patients with isolated urachal cyst infection. Presentations complicated by urinary bladder fistula, large abscesses, and sepsis are more likely to benefit from a staged approach with prolonged IV antibiotics, ultrasound-guided I&D, and surgical excision after resolution of the acute urachal cyst infection.

## Conflicts of interest

None.

## Funding

None.

## Ethical approval

This report was conducted in compliance with ethical standards.

## Consent

Informed consent has been obtained and all identifying information is omitted.

## Author contribution

Adel Elkbuli, Dessy Boneva, Kyle Kinslow, Mark McKenney– Conception of study, acquisition of data, analysis and interpretation of data.

Adel Elkbuli, Dessy Boneva, Kyle Kinslow - Drafting the article.

Dessy Boneva, Mark McKenney – Management of case.

Adel Elkbuli, Kyle Kinslow, John D. Ehrhardt Jr, Dessy Boneva, Shaikh Hai, Mark McKenney – Critical revision of article and final approval of the version to be submitted.

## Registration of research studies

This is a case report study.

## Guarantor

Dessy Boneva.

Mark McKenney.

Shaikh Hai.

## Provenance and peer review

Not commissioned, externally peer reviewed.

## References

[1] Sadler T.W., Sadler T.W. (2012). Urogenital system. Langman’s Medical Embryology.

[2] Cilley R.E., Coran A.G. (2012). Disorders of the umbilicus. Pediatric Surgery.

[3] James V., Seguin J., Kwan C.W. (2017). Urachal cyst diagnosed by point-of-care ultrasound. Clin. Pract. Cases Emerg. Med..

[4] Parada Villavicencio C., Adam S.Z., Nikolaidis P. (2016). Imaging of the urachus: anomalies, complications, and mimics. Radiographics.

[5] Ekwueme K.C., Parr N.J. (2009). Infected urachal cyst in an adult: a case report and review of the literature. Cases J..

[6] Qureshi K., Maskell D., McMillan C., Wijewardena C. (2013). An infected urachal cyst presenting as an acute abdomen - a case report. Int. J. Surg. Case Rep..

[7] Agha R.A., Borrelli M.R., Farwana R., Koshy K., Fowler A., Orgill D.P., For the SCARE Group (2018). The SCARE 2018 statement: updating consensus surgical case report (SCARE) guidelines. Int. J. Surg..

[8] Galati V., Donovan B., Ramji F. (2008). Management of urachal remnants in early childhood. J. Urol..

[9] Minevich E., Wacksman J., Lewis A.G. (1997). The infected urachal cyst: primary excision versus a staged approach. J. Urol..

[10] Masuko T., Nakayama H., Aoki N. (2006). Staged approach to the urachal cyst with infected omphalitis. Int. Surg..

[11] Yoo K.H., Lee S.J., Chang S.G. (2006). Treatment of infected urachal cysts. Yonsei Med. J..

[12] Ash A., Gujral R., Raio C. (2012). Infected urachal cyst initially misdiagnosed as an incarcerated umbilical hernia. J. Emerg. Med..

[13] Lipskar A.M., Glick R.D., Rosen N.G. (2010). Nonoperative management of symptomatic urachal anomalies. J. Pediatr. Surg..

[14] Gleason J.M., Bowlin P.R., Bagli D.J. (2015). A comprehensive review of pediatric urachal anomalies and predictive analysis for adult urachal adenocarcinoma. J. Urol..

[15] Ashley R.A., Inman B.A., Routh J.C. (2007). Urachal anomalies: a longitudinal study of urachal remnants in children and adults. J. Urol..

